# Cardiovascular autonomic testing in the work-up of cerebellar ataxia: insight from an observational single center study

**DOI:** 10.1007/s00415-019-09684-4

**Published:** 2019-12-31

**Authors:** Elisabetta Indelicato, Alessandra Fanciulli, Wolfgang Nachbauer, Andreas Eigentler, Matthias Amprosi, Jean-Pierre Ndayisaba, Roberta Granata, Gregor Wenning, Sylvia Boesch

**Affiliations:** Department of Neurology, Innsbruck Medical University, Innsbruck, Austria

**Keywords:** Cardiovascular autonomic testing, Sporadic ataxia, Multiple system atrophy of cerebellar type, Orthostatic hypotension

## Abstract

**Background:**

Cerebellar ataxias are a heterogeneous group of disorders of both genetic and non-genetic origin. In sporadic cases, two entities are recognized: multiple system atrophy of cerebellar type (MSA-C) and SAOA (sporadic adult-onset ataxia). The presence of severe cardiovascular autonomic failure reliably distinguishes MSA-C from other ataxias, but it may appear only late in the disease course.

**Objective:**

To evaluate the diagnostic yield of cardiovascular autonomic function tests in the work-up of cerebellar ataxia.

**Methods:**

We applied a cardiovascular autonomic tests battery in consecutive patients with neurodegenerative cerebellar ataxia and matched healthy controls. We recorded the presence of both orthostatic hypotension (OH) and blood pressure falls non-fulfilling the criteria of OH (non-OH BP). Sporadic cases were followed-up for an eventual conversion to MSA-C.

**Results:**

Forty-two patients were recruited, 19 of whom with sporadic disease (2 probable MSA-C, 6 possible MSA-C, 11 SAOA). Sporadic and hereditary cases showed no difference concerning ataxia severity at baseline. At head-up tilt, non-OH BP falls were detected in nine patients, but not in controls. This finding was significantly more frequent in sporadic cases (*p* = 0.006) and was detected in five out of seven patients that during follow-up converted to possible/probable MSA-C. Findings at standing test were normal in four out of nine cases with non-OH BP falls at head-up tilt.

**Conclusions:**

A complete cardiovascular autonomic battery with head-up tilt can detect early signs of BP dysregulation which may be missed at bed-side tests, thus warranting its application in the first line work-up of cerebellar ataxias.

**Electronic supplementary material:**

The online version of this article (10.1007/s00415-019-09684-4) contains supplementary material, which is available to authorized users.

## Introduction

Cerebellar ataxias are a heterogeneous group of disorders of both genetic and non-genetic origin [1–4]. Hereditary forms include a variety of entities with both autosomal dominant and recessive inheritance pattern [1, 2], the most common being the spinocerebellar ataxias type 1, 2, 3, and 6, which are caused by a CAG triplet expansion in the respective loci [2]. In several patients with adult-onset ataxia a specific genetic cause cannot be found, suggesting a diagnosis of sporadic degenerative ataxia [4]. In such context, the best characterized disease entity is represented by multiple system atrophy (MSA) [5]. MSA is a fatal progressive disorder featuring a variable combination of Parkinsonism, autonomic dysfunction, cerebellar and pyramidal signs [5]. It is classified as MSA-parkinsonian (MSA-P), if Parkinsonism prevails or MSA-cerebellar (MSA-C) if cerebellar features predominate. Sporadic adult-onset ataxias not being diagnosed as MSA-C are defined as SAOA (simply “sporadic adult-onset ataxia”) [4]. SAOA is a clinically defined entity since neuropathological study are lacking. The essential difference between SAOA and MSA-C is given by absence of severe autonomic failure in the first [4, 6]. In SAOA, cerebellar features may be accompanied by other neurological findings such as sensory deficits and pyramidal signs [4, 7]. While SAOA generally shows a benign disease course, MSA-C is a severe and rapidly progressive disorder, especially if overt dysautonomia develops early in the disease course [5, 8]. According to an earlier study [9], up to 25% of SAOA convert to MSA-C later on.

In this light, cardiovascular autonomic function tests (CAFTs) are integral part of the diagnostic work-up of cerebellar ataxia [7, 10]. CAFTs allow the detection of orthostatic hypotension (OH), namely a sustained BP drop ≥ 20 mmHg systolic or ≥ 10 mmHg diastolic or an absolute systolic BP value < 90 mmHg after 3 min of head-up tilt or active standing, and ascertain its neurogenic origin [11, 12]. Hereditary cerebellar ataxias and SAOA may experience various autonomic symptoms [7, 13, 14], but orthostatic hypotension is rare [15] and its detection enables the diagnosis of “possible MSA-C”, according to international criteria [6]. A severe orthostatic hypotension, namely a BP fall > 30 mmHg systolic or > 15 mmHg diastolic is required to diagnose “probable” MSA-C [6].

Although OH is very specific for MSA, it may be a late feature in its cerebellar variant [9], implying difficulties in early diagnosis and management. Differently from MSA-P, only few studies focused on the differential diagnosis of MSA-C and longitudinal data on sporadic ataxias are limited.

In the present study, we performed (1) a cross-sectional autonomic evaluation including standardized cardiovascular tests in patients with cerebellar ataxia referring to our Department and (2) followed-up patients with sporadic ataxia for eventual conversion to MSA-C. Our aim was to evaluate the diagnostic yield of cardiovascular autonomic function tests in the work-up of sporadic cerebellar ataxia and possibly identify early markers of cardiovascular autonomic failure.

## Patients and methods

### Participants

We screened patients with cerebellar ataxia during inpatient stay from January 2016 until December 2017. We included patients with hereditary cerebellar ataxia and sporadic cerebellar ataxia not secondary to other causes (vascular, neoplastic, toxic, metabolic, infectious or autoimmune causes of cerebellar pathology including positive anti-GAD, anti-NMDA and onconeural antibodies). Sporadic ataxia was diagnosed if the further following criteria were met: onset > 30 years old, negative and informative family history, negative testing for SCA 1, 2, 3, 6 FRDA, no attack-like symptoms [7, 16, 17]. Possible or probable MSA-C was diagnosed according to Gilman criteria [6]. Sporadic ataxia cases which did not meet criteria for MSA-C were diagnosed as SAOA.

Cardiac arrhythmias, symptomatic heart disease (NYHA ≥ 2) as well as secondary causes of autonomic dysfunction (impaired glucose metabolism, vitamin B12 or folate deficiency, non-compensated thyroid disease, amyloidosis, connective tissue disease, etc.) or of non-neurogenic orthostatic hypotension (medications such as alpha blockers or vasodilating agents, anemia, dehydration) were set as further exclusion criteria. Patient between 18 and 80 years of age were considered eligible.

A comparison with age- and sex-matched healthy controls from our laboratory was performed.

### Clinical scales

Within a general and neurological examination, standardized clinical scales were administered. We applied the scale for the assessment and rating of ataxia (SARA) as a measure of ataxia severity [18]. The SARA consist of 8 items for a final score between 0 (= no ataxia) and 40 (= the most severe ataxia, bed ridden). An MSA specific scale, the UMSARS (unified multiple system atrophy rating scale), was also applied to all patients as a broader neurological evaluation tool [19]. The subscale UMSARS I evaluates functional disabilities and autonomic symptoms burden, while the UMSARS II is a motor examination considering both extrapyramidal and cerebellar signs. Lastly, we administered two validated autonomic questionnaire, the SCOPA-Aut (autonomic scale for outcomes in Parkinson's disease) [20], assessing a broad spectrum of autonomic symptoms, and the Orthostatic Hypotension Questionnaire (OHQ) about cardiovascular autonomic symptoms [21]. Findings from the urogenital domain of SCOPA-Aut in both sporadic and hereditary cases are detailed reported in the results.

### Cardiovascular autonomic function tests: performance

CAFTs were performed in a quiet room at a constant temperature following a standardized protocol of our laboratory, which has been described in detail elsewhere [22]. CAFTs included the following procedures: 10 min supine rest, 10 min 60° Head-up tilt, 5 min supine rest, 5 min of active standing, deep breathing, and Valsalva maneuver. Heart rate (HR) and BP were continuously recorded using a CNSystem device (CNSystem Medizintechnik GmbH, Graz, Austria). Biosignals’ analysis was performed by means of a customized software (by JPN).

### Cardiovascular autonomic function tests: evaluation

OH was diagnosed according to the abovementioned consensus criteria [12, 23]. The neurogenic nature of OH was corroborated by detection of blunted/absent increase in heart rate upon tilting and absence of BP overshoot in the phase IV of valsalva maneuver [24]. Even in absence of OH, a number of signs at CAFTs may suggest incipient autonomic failure. These consist of (1) impaired HR counter-regulation during deep breathing and (2) valsalva maneuver [25] as well as (3) delayed OH. We also considered as further possible sign of incipient dysautonomia (4) falls of BP at 3 min of orthostatic challenge, which did not meet the requirements for diagnosis of OH. Methodological details are reported in Table 1.

**Table 1:** Additional cardiovascular autonomic function indices: methodological details and references

Impaired deep breathing	Reduced deep breathing ratio compared to age- and sex-matched healthy controls of our lab [22, 25]
Impaired valsalva maneuver	Reduced Valsalva ratio compared to age- and sex-matched healthy controls of our lab [22, 25]
Delayed orthostatic hypotension	BP fall > 20 mmHg systolic or > 10 mmHg diastolic occurring later than 3 min after begin of the orthostatic stress [26]
Non-OH BP falls	BP falls at 3 min of head-up tilt or active standing < 20 mmHg systolic or < 10 mmHg diastolic

### Follow-up

Patients where regularly followed-up in 3–12 months intervals. Sporadic cases were monitored concerning an eventual conversion to possible/probable MSA-C.

### Statistical analysis

Statistical analysis was performed with SPSS version 25. Categorical variables are reported as percentages and continuous variables either as mean ± SD or median (interquartile range) according to their distribution. Normality of distribution was tested by means of Shapiro–Wilk test. Group comparisons were performed between (a) patients and controls or (b) hereditary vs sporadic cases either by means of *t* test or Mann–Whitney *U* test, according to variable distribution. Statistical significance was set at *p* < 0.05.

## Results

### Demographic and baseline clinical data

Forty-two patients were recruited, of whom 18 (43%) at the time of their first referral. Nineteen patients (45%) were diagnosed as having sporadic cerebellar ataxia, with *n* = 6 and *n* = 2 meeting the criteria of possible and probable MSA-C at baseline. Mean age at examination was 53 ± 13 years. Median disease duration was 10 (3; 15) years. As expected, patients with sporadic ataxia had significantly later disease onset (53 ± 8 vs 33 ± 15, *p* = 0.003) and had shorter disease duration [3 (2; 14) vs 13 (7; 19) *p* = 0.008] at baseline compared to hereditary cases. Severity of ataxia as expressed by SARA scores did not differed between the two groups. Scores at UMSARS I and SCOPA-Aut, expressing functional disability and autonomic symptoms burden, were higher in patients with sporadic disease (see Table 2). Within the sporadic group, the only score that significantly differed between MSA-C and SAOA cases was SCOPA-Aut (MSA-C = 15 ± 5, SAOA = 8.5 ± 5, *p* = 0.01, see also supplementary material).

**Table 2:** Clinical-demographic characteristics at baseline in the whole cohort and in the hereditary/sporadic subgroups

	Whole cohort (*n* = 42)	Hereditary (*n* = 23)	Sporadic (*n* = 19)	Statistic
Age at examination	53 ± 13	47 ± 12	61 ± 10	0.67
Sex (females, %)	13 (31%)	6 (25%)	7 (39%)	0.52
Age at onset	46 (28;56)	33 ± 15	53 ± 8	**0.003**
Disease duration	10 (3;15)	13 (7;19)	3 (2;14)	**0.008**
Death during follow-up (no. of cases)	2 (4.7%)	0	2 (10.5%)	0.2
SARA	13.5 (9;17.5)	14 ± 7	14 ± 5	0.08
UMSARS I	7.5 (5;12)	5 (3;10)	9 (6;14)	**0.04**
UMSARS II	16.3 ± 7.4	12 (10;19)	19 (12;21)	0.29
SCOPA-Aut	7 (3;14)	4 (3;12)	12 (7;15)	**0.03**
OHQ	0 (0;4)	0 (0;2.5)	0 (0;5.3)	0.32

Statistically significant differences are reported in bold

### Findings at baseline CAFTs

Due to neurological disabilities the head-up tilt and the active standing test were performed in 36 and 39 patients out of 42, respectively. Findings in the hereditary group were largely comparable with those of healthy controls, except for a higher rest hear rate and lower deep breathing ratio in patients. Conversely, sporadic cases showed significant differences from controls in all tests (see Tables 1 and 2 in supplementary material). At head-up tilt, 3 patients had OH (2 probable MSA-C, 1 possible MSA-C). Further 2 patients had delayed OH (1 possible MSA-C, 1 SAOA). At 3 min head-up tilt, 10 patients showed BP falls non-fulfilling the OH criteria (28%, 4 possible MSA-C, 4 SAOA and 2 hereditary cases). This finding was detected in none of the controls (*p* = 0.0004 in the comparison sporadic cases vs controls) and was significantly more frequent in the sporadic group (*p* = 0.006 in the comparison sporadic *vs* hereditary cases). The two hereditary cases displaying non-OH BP falls were one with autosomal recessive ataxia (woman, Age = 65, SARA = 17, disease duration = 13) and one with spinocerebellar ataxia type 2 (man, age = 31, SARA = 25, disease duration = 12). Both had pons atrophy at MR imaging. In sporadic patients with non-OH BP fall, mean systolic BP change at 3 min of head-up tilt was − 5.7 ± 4 mmHg and median diastolic change − 1 (− 4;10) mmHg. An increase in heart rate upon tilt was still detected in all of them (mean HR change 17 ± 7 beats/min) but in four out of seven cases BP overshoot in phase IV of Valsalva Maneuver was missing.

In four out of nine patients with non-OH-BP fall at head-up tilt, findings at 3 min of active standing were normal.

One patient could not perform Valsalva maneuver due to severe limb ataxia. In further cases (*n* = 4 at Valsalva maneuver, *n* = 8 at deep breathing), test was performed, but artifacts due to motor disabilities precluded its analysis. Valsalva ratio was reduced in 24 patients (65%); deep breathing ratio was reduced in 26 patients (74%). Blunted HR response at deep breathing and Valsalva was more frequent in sporadic cases (see also Table 3 and supplementary material for row data).

**Table 3:** Findings at CAFTs

	All cohort	Hereditary	Sporadic	Statistic
OH	3/36 (7%)	0/19	3/17 (18%)	0.1
Delayed OH	2/33 (6%)^a^	0/19	2/14 (14%)^a^	0.2
Non-OH BP falls	10/33 (30%)^a^	2/19 (10%)	8/14 (57%)^a^	**0.006**
Impaired deep breathing	26/35 (74%)	13/19 (68%)	13/16 (81%)	0.3
Impaired Valsalva maneuver	24/37 (65%)	10/20 (50%)	14/17 (82%)	**0.04**

Statistically significant differences are reported in bold

^a^Patients with OH where excluded from this analysis

### Urogenital symptoms at baseline

Twenty-eight patients (67%) reported urogenital symptoms at SCOPA-Aut (see also supplementary material). Sporadic cases had generally higher frequency of urogenital symptoms, although no significant differences was found concerning frequency of urinary symptoms, sexual symptoms and use of medications for urogenital symptoms compared to hereditary cases (see also Table 5 in supplementary material). All MSA-C cases (8 out of 8) and the majority of SAOA patients (9 out of 11) reported urogenital symptoms (*p* = 0.3 in the comparison). The only two SAOA without urogenital symptoms at baseline were male patients who in the follow-up developed erectile dysfunction and therefore classified as converters (see next paragraph).

### Follow-up

Eighteen out of 19 patients with sporadic ataxia were regularly followed-up. Median follow-up was 15 (12; 33) months. One patient (one probable MSA-C) died during the follow-up. Of six possible MSA-C, three converted to probable MSA-C and one of them died shortly after conversion. All three converters had non-OH BP fall at baseline CAFTs. Out of 11 SAOA, 1 was lost at follow-up and 4 (36%) converted to possible MSA-C. Time to conversion from baseline was 24, 9, 9 and 7 months respectively. Conversion occurred between 31 and 69 months after disease onset. In 2 of these cases, conversion was established due to onset of OH; notably they had > 2 abnormal autonomic indexes at baseline CAFTs (including non-OH BP fall). In the other two converters, possible MSA-C was established because of development of erectile dysfunction during follow-up. They had largely normal CAFTs findings. In two other SAOA (18%) a genetic variant of unknown significance was detected in a subsequent exome wide analysis. These two cases had also normal findings at CAFTs. At baseline, converters to MSA-C had lower scores at both motor and autonomic scales compared to SAOA who did not convert (not significant in statistical comparison, see also supplementary material).

## Discussion

In the present study, we applied CAFTs in a cohort of patients with hereditary and sporadic neurodegenerative cerebellar ataxia referring to our inpatient clinic. CAFTs detected OH exclusively in patients with sporadic ataxias, which were therefore diagnosed as having possible/probable MSA-C. Beyond that, BP falls during orthostatic stress could be detected in several patients, not being sufficient to diagnose OH or probable MSA-C according to the current criteria. Interestingly this finding was significantly associated with sporadic disease and was present in five out of seven patients who later converted to possible/probable MSA-C. The physiological response to orthostatic stress consists of peripheral vasoconstriction and increase in heart frequency to counteract the venous pooling induced by gravity. These responses aim at preventing blood pressure drop and results in maintenance of systolic and slight increase of diastolic blood pressure values [27]. In this light, also minor BP falls upon orthostatic challenge may indicate an incipient impairment in the physiological BP counter-regulation. It appears unlikely that the observed non-OH BP falls in the far older sporadic cases simply reflect an age-related change since this finding was not observed in the controls, but in one young patient with hereditary ataxia. Interestingly this finding was detected also in two hereditary cases, with different etiologies and age at examination. Both displayed pons involvement, but also had long standing disease and more severe ataxia compared to the sporadic cases.

Notably, the standing test was normal in four out of nine patients with non-OH BP falls at head-up tilt, pointing out the higher sensitivity of a comprehensive battery of cardiovascular autonomic function tests in a dedicated laboratory in the differential diagnostics of cerebellar ataxia. Impairment in Valsalva and deep breathing were frequent in SAOA patients, but were also recurrently detected in hereditary ataxia cases and, more importantly, in this cohort of patients with cerebellar syndrome were frequently non analyzable due to motor disabilities related artifacts.

As expected, sporadic cases had older age at presentation and shorter disease duration. Though, sporadic and hereditary cases did not differ at presentation concerning severity of ataxia or other motor symptoms as determined by SARA and UMSARS II scores. The only significant difference in basal clinical data was found in the autonomic and functional disability scores (SCOPA-aut, UMSARS I), which were generally higher in the sporadic group. An effect of age cannot be excluded here.

SAOA is a clinically defined entity including late onset ataxias not fulfilling MSA-C criteria and its neuropathological substrate remains elusive. At baseline, milder cardiovascular autonomic impairment and higher autonomic scores were present in the sporadic ataxia group as a whole compared to hereditary cases.

During follow-up, 18% of SAOA patients turned out to have a genetic diagnosis and 36% converted to possible MSA-C. Altogether more than 50% of SAOA cases turned out to have another diagnosis during follow-up and other 50% of possible MSA-C at baseline progressed to probable MSA. Our data is in line with current research showing that an increasing percentage of SAOA cases can be reclassified as genetically determined [7, 28]. Importantly, Cortese et al. recently demonstrated that biallelic expansion of an intronic repeat in *RFC1* gene can underlie up to 22% of SAOA cases [28], thus further reducing the percentage of the “unsolved” SAOA. Notably, in the study by Cortese et al. several cases reclassified as hereditary showed autonomic disturbances such as urinary problems or erectile dysfunction, while “impaired regulation of BP” applied to only one patient [28]. Also in our cohort, urogenital symptoms were highly frequent also in hereditary cases and SAOA who did not convert to MSA-C. These findings suggest that cardiovascular autonomic failure still remains the most specific sign pointing towards a diagnosis of MSA-C in the setting of a sporadic adult-onset ataxia, thus strengthening the diagnostic role of CAFTs in the genetic era.

Our study is limited to a single center and a small casuistic. Thus, we strengthened our work by applying strict inclusion criteria and a detailed patient characterization/evaluation in a field where clinical research is still limited. SAOA and MSA are rare disorders and MSA-C is particularly uncommon in the Caucasian population compared to his parkinsonian variant [5]. Nonetheless, in this study conducted at a specialized ataxia unit, sporadic cases accounted for approximately the half of the whole cohort. We did not screen our patients for *RFC1* expansions because they were described after completion of the study. Up to date, only few studies addressed CAFTs in the differential diagnosis of sporadic ataxias. These were performed before the availability of actual genetic testing and considered younger patients collectives, thus being likely of including a large proportion of genetically determined cases. Only recently, a European consortium established a registry on sporadic ataxia to systematically address the diagnostic dilemma between MSA-C and SAOA, but did not consider CAFTs examination [7].

In conclusion, the present study supports the utility of CAFTs and standardized autonomic history as first line examination in patient with adult-onset cerebellar ataxia with negative family history. Further studies in independent, larger cohort are advocated to confirm our findings. Absence of milder signs of cardiovascular autonomic failure may suggest a non-sporadic disorder and direct to second line genetic testing.

## Electronic supplementary material

Below is the link to the electronic supplementary material.

Supplementary file1 (DOCX 25 kb)
