# Supplementary material for: Cardiovascular autonomic testing in the work-up of cerebellar ataxia: insight from an observational single center study
Source: J Neurol. 2019 Dec 31;267(4):1097–102. doi: 10.1007/s00415-019-09684-4 (PMC7109187; doi:10.1007/s00415-019-09684-4)
Supplement: Supplementary file 1 — Supplementary file1 (DOCX 25 kb) [file 415_2019_9684_MOESM1_ESM.docx]

**Supplementary material**

**Supplementary Table 1 - Cardiovascular autonomic function testing in hereditary ataxia cases *vs* age- and gender-matched healthy controls.**

| **Test** | **Hereditary ataxia group** | | **Controls** | | **p** |
| --- | --- | --- | --- | --- | --- |
| 10 min. supine rest | N=19 | | N=19 | |  |
| heart rate | 68±11 | | 60±7 | | ***0,01*** |
| systolic BP | 114±11 | | 117±10 | | n.s. |
| diastolic BP | 75±9 | | 77±9 | | n.s. |
|  |  |  | |  | |
| Δ at 3 min. head-up tilt | N=19 | | N=19 | |  |
| heart rate | +11±6 | | +11±6 | | n.s. |
| systolic BP | +9±8 | | +11±6 | | n.s. |
| diastolic BP | +13±7 | | +12±5 | | n.s. |
|  |  | |  | |  |
| Δ at 10 min. head-up tilt | N=19 | | N=19 | |  |
| heart rate | +15±10 | | +13±8 | | n.s. |
| systolic BP | +5±9 | | +9±7 | | n.s. |
| diastolic BP | +9±7 | | +11±7 | | n.s. |
|  |  | |  | |  |
| Δ at 3 min. active standing | N=21 | | N=21 | |  |
| Heart rate | +17(13;22) | | +17(13;25) | | n.s. |
| systolic BP | +12±10 | | +14±12 | | n.s. |
| diastolic BP | +15±9 | | +19±10 | | n.s. |
|  |  | |  | |  |
| Δ at 5 min. active standing | N=19 | | N=19 | |  |
| Heart rate | 16(14;23) | | 18(10;23) | | n.s. |
| systolic BP | +10±8 | | +12±10 | | n.s. |
| diastolic BP | +8±8 | | +15±7 | | n.s. |
|  |  | |  | |  |
| Deep Breathing Ratio | N=19 | | N=19 | |  |
|  | 13(8;17) | | 20(15;24) | | ***0,01*** |
|  |  | |  | |  |
| Valsalva Maneuver | N=20 | | N=20 | |  |
| Valsalva Ratio | 1,65±0,4 | | 1,8±0,3 | | n.s. |
|  |  | |  | |  |

**Supplementary Table 2 - Cardiovascular autonomic function testing in sporadic ataxia cases *vs* age- and gender-matched healthy controls.**

| **Test** | **Sporadic ataxia group** | | **Controls** | | **p** |
| --- | --- | --- | --- | --- | --- |
| 10 min supine rest | N=17 | | N=17 | |  |
| heart rate | 69±12 | | 58±6 | | ***0,006*** |
| systolic BP | 116±16 | | 121±11 | | n.s. |
| diastolic BP | 74±11 | | 79±7 | | n.s. |
|  |  |  | |  | |
| Δ at 3 min. head-up tilt | N=17 | | N=17 | |  |
| heart rate | +13±7,8 | | +11,9±8 | | n.s. |
| systolic BP | -6±13 | | +10±5 | | ***0,00002*** |
| diastolic BP | +0,5±12 | | +12±5 | | ***0,001*** |
|  |  | |  | |  |
| Δ at 10 min. head-up tilt | N=17 | | N=17 | |  |
| heart rate | +12±9 | | +13±6 | | n.s. |
| systolic BP | -7±17 | | +8±6 | | ***0,002*** |
| diastolic BP | -1±13 | | +10±5 | | ***0,006*** |
|  |  | |  | |  |
| Δ at 3 min. active standing | N=15 | | N=15 | |  |
| Heart rate | +17,3±7,5 | | +19,5±6,7 | | n.s. |
| systolic BP | +3(-4;11) | | +15(4;19) | | ***0,02*** |
| diastolic BP | +17,9±9,5 | | +22,3±14,4 | | ***0,008*** |
|  |  | |  | |  |
| Δ at 5 min. active standing | N=14 | | N=14 | |  |
| Heart rate | 14(12;17) | | 16(10;25) | | n.s. |
| systolic BP | +5±11 | | +11±12 | | n.s. |
| diastolic BP | +9±7 | | +15±9 | | n.s. |
|  |  | |  | |  |
| Deep Breathing Ratio | N=16 | | N=16 | |  |
|  | 10±4 | | 16±7 | | ***0,01*** |
|  |  | |  | |  |
| Valsalva Maneuver | N=17 | | N=17 | |  |
| Valsalva Ratio | 1,27(1,14;1,48) | | 1,65(1,15;1,9) | | ***0,002*** |
|  |  | |  | |  |

**Supplementary Table 3 – Clinical scales of patients with baseline diagnosis of multiple system atrophy of cerebellar type (MSA-C) versus sporadic adult onset ataxia (SAOA).** SARA: Scale for Assessment and Rating of Ataxia; UMSARS: Unified Multiple System Atrophy Rating Scale; SCOPA-Aut: Scale for outcomes in Parkinson’s Disease; OHQ: Orthostatic Hypotension Questionnaire.

|  | **MSA-C (n=8)** | **SAOA (n=11)** | **Statistic** |
| --- | --- | --- | --- |
| SARA | 14±4 | 14±5 | 0,98 |
| UMSARS I | 11,5±5 | 9±5 | 0,4 |
| UMSARS II | 17±6 | 17±8 | 0,9 |
| SCOPA-Aut | 15±5 | 8,5±5 | ***0,01*** |
| OHQ | 5 (2;7) | 0 (0;5) | 0,2 |

**Supplementary Table 4 – Baseline clinical scales of patients, who later converted to multiple system atrophy of cerebellar type versus patients retaining the diagnosis of sporadic adult onset ataxia.** SARA: Scale for Assessment and Rating of Ataxia; UMSARS: Unified Multiple System Atrophy Rating Scale; SCOPA-Aut: Scale for outcomes in Parkinson’s Disease; OHQ: Orthostatic Hypotension Questionnaire.

|  | **Converters**  **(4)** | **Non-Converters (6)** | **Statistic** |
| --- | --- | --- | --- |
| SARA | 10±3 | 15±6 | 0,1 |
| UMSARS I | 7±2 | 10±7 | 0,4 |
| UMSARS II | 12±7 | 19±8 | 0,3 |
| SCOPA-Aut | 5±6 | 10±4 | 0,4 |
| OHQ | 0 (0;3,5) | 0 (0;5,5) | 1 |

**Supplementary Table 5 – Urogenital symptoms in patients with hereditary versus sporadic adult onset ataxia.**

|  | **Hereditary** | **Sporadic** | **Statistic** |
| --- | --- | --- | --- |
| Urinary symptoms | 17/23 (74%) | 17/19 (89%) | 0,2 |
| Sexual Dysfunction* | 5/20* (25%) | 6/14* (43%) | 0,2 |
| Medications for urogenital symptoms | 1/23 (4%) | 1/19 (5%) | 0,7 |

* Data available for 34 out of 42 patients for this item (81% of the study population).

**Supplementary Table 6 – Urogenital symptoms in patients with a baseline diagnosis of multiple system atrophy of cerebellar type (MSA-C) versus sporadic adult onset ataxia (SAOA).**

|  | **MSA-C** | **SAOA** | **Statistic** |
| --- | --- | --- | --- |
| Urinary symptoms | 8/8 (100%) | 9/11 (82%) | 0,3 |
| Sexual Dysfunction* | 3/6* (50%) | 3/8* (38%) | 0,5 |
| Medications for urogenital symptoms | 1/8 (13%) | 0/11 (0%) | 0,4 |

* Data available for 14 out of 19 patients for this item (74% of the study population).
